# Transcorneal Permeation of Ciprofloxacin and Diclofenac from Marketed Eye Drops

**DOI:** 10.4103/0250-474X.45409

**Published:** 2008

**Authors:** N. Patidar, M. S. Rathore, D. K. Sharma, A. Middha, V. B. Gupta

**Affiliations:** Department of Pharmaceutics, B.R. Nahata College of Pharmacy, Mhow Neemuch Road, Mandsaur-458 001, India

**Keywords:** Diclofenac, ciprofloxacin, goat cornea, transcorneal permeation, eye drops

## Abstract

The purpose of this research was to evaluate the *in vitro* permeation characteristics of various marketed eye drops of ciprofloxacin (0.3% w/v aqueous solution) and diclofenac (0.1% w/v aqueous solution) through isolated goat cornea. Effect of these drugs on isolated goat eye lenses was also evaluated. Permeation studies were conducted by putting 1 ml of formulation on the cornea fixed between the donor and receptor compartments of an all glass modified Franz diffusion cell and monitoring ciprofloxacin and diclofenac concentration in the receptor (containing normal saline or bicarbonate ringer solution under continuous stirring at 37±2°) spectrophotometrically at their respective absorption maxima, after 120 min. Paired isolated goat lenses (i.e. of same animal) were used to evaluate the effect of these drugs at selected concentrations against oxidative stress (1 mM hydrogen peroxide solution). After 24 h of incubation at 37°, the lens treated with test solution (hydrogen peroxide+drug in bicarbonate ringer solution) was estimated for soluble protein content and compared with control (only hydrogen peroxide). Among marketed eye drops of ciprofloxacin, Joxin (Jawa Pharmaceuticals) showed maximum *in vitro* transcorneal permeation (0.558%) while I-Gesic (Centaur Pharmaceuticals) showed maximum % *in vitro* permeation or *in vitro* ocular availability among diclofenac eye drops after 120 min of permeation. The soluble protein content estimation studies revealed that these drugs at selected concentrations (permeated after 120 min.) had no deleterious effect on eye lenses rather possessed protective effect, since all formulation showed more soluble protein content when compared with control.

Eye drops of ciprofloxacin and diclofenac have proven their efficacy in conjunctivitis, keratitis, and endophthalmitis^1^–^5^. Based on the benefits offered by these drugs various manufactures have introduced eye drops of ciprofloxacin (0.3% w/v) and diclofenac (0.1% w/v). The available eye drops, being manufactured by different manufactures may vary in the type/concentration of additives and thereby may have varying permeation characteristics through cornea. Some drugs are known to cause cataract development (opacity of lens)^6^ while some have shown protective effects on lens against cataract development^7^.

Based on the above facts this study was designed to evaluate *in vitro* permeation characteristics of marketed eye drops of ciprofloxacin and diclofenac through isolated goat cornea. In addition efforts were made to know the deleterious or protective effect of these eye drops on isolated goat eye lens under oxidative stress conditions.

Ciprofloxacin HCl and diclofenac sodium were generous gift samples from Asoj Soft Caps Pvt. Ltd., Vadodara. Marketed eye drops Joxin (Jawa Pharmaceutical), Cinfax (Optho Remedies), Ceprolen (Indoco Remedies), Cifran (Ranbaxy), Ciplox (Cipla), Diclolab (Laborate Pharmaceuticals), NS-AID (Syntho Pharmaceuticals), I-Gesic (Centaur Pharmaceuticals) were procured from local medicine market. All chemicals were of analytical grade and were used as received. Fresh whole eyeballs of goat were collected from local butcher' shop (Sanjeet Naka, Mandsaur) within an hour of slaughtering the animal.

One millilitre of each marketed formulation of ciprofloxacin was titrated with 0.1 N NaOH to phenolphthalein end point to evaluate the buffering capacity of the preparation. *In vitro* permeation studies were carried out in an all glass modified Franz diffusion cell containing a donor and receptor cells as described by Malhotra and Majumdar^8^. The donor cell was clamped over the receptor, which was provided with a side arm for sampling and had an internal capacity of 11 ml. The area available for the corneal transport was 0.785 cm^2^. The receptor contained normal saline or bicarbonate ringer solution.

The preparation of cornea and experimental procedure was same as described in the literature^8^. For estimation of soluble protein content (SPC) incision on goat eyeball was made (on scleral portion) and lens was taken out carefully with intact capsule then washed with normal saline. The lenses of the same animal were incubated for 24 h at 37° in either Mg free Tyrode medium 10 ml containing 1 mM H_2_O_2_ or Mg free Tyrode medium 10 ml containing 1 mM H_2_O_2_ with solution under test. Following the specified period of incubation the lenses were taken out from the solution and washed with normal saline. Each lens was decapsulated and homogenized in 2 ml of 0.9% normal saline. The homogenates were centrifuged at 6000 rpm for 1 h. The supernatant was analyzed for protein concentration using Folin-Lowry method of protein assay^9^.

Permeation characteristics of five marketed eye drops of ciprofloxacin (0.3% w/v) were evaluated for permeation characteristics through excised goat cornea. According to permeation studies Joxin (Jawa Pharmaceutical) showed maximum% permeation (0.558%) or *in vitro* ocular availability followed by Cinfax (0.542%) and Ceprolen (0.156%) while Cifran (0.117%) and Ciplox (0.118%) showed almost similar % permeation after 120 min through excised goat cornea. Cinfax and Joxin showed corneal hydration lower than normal range (75-80%) and rest of formulations were having corneal hydration in normal range. Results are shown in Table 1.

**TABLE 1:** RELATIVE PERMEATION CHARACTERISTICS OF CIPROFLOXACIN FROM MARKETED EYE DROPS THROUGH EXCISED GOAT CORNEA

Brand name	Amount permeated through cornea after 120 min (mg)	Permeation (%) (120 min)	Corneal hydration (%) 120 min	pH of ophthalmic solution	Volume used of 0.1 N NaOH (ml)
Cifran	0.00353±0.00014	0.117±0.0046	76.6±0.638	4.03±0.088	0.233±0.033
Ceprolen	0.0047±0.00015	0.156±0.0051	77.7±0.288	3.86±0.066	1.33±0.033
Cinfax	0.0162±0.00148	0.542±0.0499	73.6±1.299	4.23±0.145	1.46±0.033
Joxin	0.0167±0.00067	0.558±0.0224	73.9±0.132	4.43±0.120	1.56±0.033
Ciplox	0.0035±0.00014	0.118±0.0046	77.8±0.490	4.3±0.115	0.266±0.033

Values are mean±SEM of 3 experiments in each group

Benzalkonium chloride (BAC) is a quaternary ammonium compound used very frequently in eye drops for primary purpose of maintaining sterility of formulation during use, since most eye drops are packed in multiple dose containers. BAC is known to increase permeation of drug by disruption of corneal epithelium^10^. The lower permeation of Cifran and Ciplox might be attributed to lower concentration of BAC (0.01%) in formulation. Joxin and Cinfax containing 0.02% concentration of BAC and showed higher % permeation. Ciprofloxacin is a weakly basic drug and at lower pH it would remain in ionized state, which is unfavorable for its permeation through cornea Ceprolen had 0.02% BAC concentration but comparatively low pH (3.86), of formulation might attribute for its lower permeation. All formulations were having corneal hydration in normal range except Cinfax and Joxin. Lower hydration level (73.6 and 73.9) showed slight hypertonicity of the formulations. Cinfax and Joxin also seem to be buffered eye drops since these consumed more volume of 0.1 N NaOH comparatively. Lower hydration level and buffered nature of these eye drops showed slight hyper tonicity and their possible irritation to eye. These eye drops will cause more tears to bring the pH equilibrate with tears (i.e. pH 7.4). Excess lacrimal fluid/tear production would cause loss of drug from precorneal space and subsequently lesser bioavailability *in vivo*. Cifran and Ciplox showed least permeation but were having comparative higher pH and were non buffered since these consumed lesser volume of 0.1 N NaOH. Ceprolen, however seems buffered (1.33 ml) and showed intermediate % permeation while corneal hydration was in normal range.

Diclofenac sodium eye drops are widely used to treat the inflammation of eye caused by several reasons (infection, trauma). Three marketed eye drops (0.1% w/v of diclofenac sodium) were evaluated for their *in vitro* permeation characteristics through excised goat cornea. I-Gesic produced maximum % permeation or *in vitro* ocular availability (5.73%) after 120 min of permeation. Another eye drop marketed under brand name Diclolab showed 5.32% permeation followed by NS-AID 2.82%, (Table 2). All eye drops were having corneal hydration under normal range except Diclolab. Diclolab showed 70.2% corneal hydration, which indicates hypertonicity of the formulation and possible irritation to eye when administrated topically.

**TABLE 2:** RELATIVE PERMEATION CHARACTERISTICS OF DICLOFENAC FROM MARKETED EYE DROPS THROUGH EXCISED GOAT CORNEA

Brand name	Amount permeated through cornea after 120 min (mg)	Permeation (%) (120 min)	Corneal hydration (%) 120 min	pH of ophthalmic solution
I-Gesic	0.0573±0.00087	5.73±0.087	76.1±0.502	5.46±0.088
Diclolab	0.0532±0.0019	5.326±0.195	70.2±0.663	6.86±0.033
NS-AID	0.0282±0.00096	2.82±0.096	78.0±0.409	6.53±0.088

Values are mean±SEM of 3 experiments in each group

Diclofenac is an acidic (anionic) drug and according to Henderson Hasselbalch equation, pH of formulation towards acidic side would be favorable for its permeation. Higher permeation of I-Gesic might be due to it comparatively lower pH (5.4). Diclolab had pH 6.8 and should show lower permeation but showed comparatively higher permeation to NS-AID, which had almost similar pH. Diclolab's higher permeation at higher pH indicates that manufacturer might have added some excipients, which caused damage to corneal epithelium (revealed by decreased hydration level, Table 2) and consequently increased the % permeation. Another eye drop named NS-AID showed least permeation among all eye drops contains Diclofenac sodium (0.1% w/v). The reason probably is addition of benzalkonium chloride as a preservative in the formulation by the manufacturer. It is mentioned in the literature^11^ that BAC (a cation) leads to a number of incompatibilities with negative charged molecules (diclofenac is negative charged species).

The protective/damaging effects of eye drops at selective concentrations (i.e. concentration that permeated after 2 h through isolated goat cornea) were evaluated using paired isolated goat eye lenses. By paired lenses, we mean the two lenses of the same animal were used. After the experiment both lenses were evaluated for total soluble protein content using Lowry's method. Results are shown in fig. 1 The drug that protects the lenses from oxidative stress will render the total soluble protein content more than the control one, while the drug that damage the lens will render total soluble protein content lesser than that of control. All formulations showed non deletrious effect on lenses at selected (i.e. at concentration/amount permeated after 120 min).

**Fig. 1:**
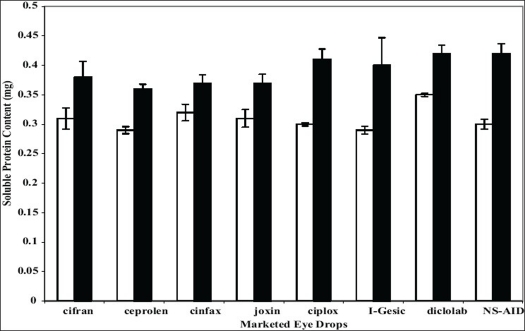
Comparative effect of 1 mM H_2_O_2_ alone and in combination with eye drops on isolated paired goat eye lenses Comparative effect on isolated paired goat eye lenses with 1 mM H_2_O_2_ solution alone (control-□) and in combination with marketed eye drop (Test-▪). Values are mean±SEM of 3 experiments in each group

On the basis of above *in vitro* studies performed using isolated goat cornea it may be concluded that among selected formulations Joxin (Jawa Pharmaceutical) eye drop showed maximum *in vitro* % permeation of ciprofloxacin while I-Gesic showed maximum % permeation or *in vitro* ocular availability of diclofenac. *In vitro* studies conducted on isolated goat eye lenses revealed the fact that all eye drops of ciprofloxacin and diclofenac are non-damaging to lenses at selected concentrations, rather these showed protective effect on isolated goat eye lenses. However, further *in vivo* studies are needed to comment more in this respect.
